# Aβ-42 Side Chain Deamidation at Q15 or N27 Modulates Protein Aggregation and Alters Microglial Cytokines and CD68

**DOI:** 10.1021/acschemneuro.6c00024

**Published:** 2026-09-08

**Authors:** Martin N. Griffin, Daniel M. Dinakarapandian, Chenxing Li, Pranay Ramaswamy, Sia J. Saheba, Janie Lee, Tarunya Rao Sudarshan, Maria Sajimon, Christopher J. Wheeler, Jevgenij A. Raskatov, Anant K. Paravastu, Levi B. Wood

**Affiliations:** † George W. Woodruff School of Mechanical Engineering, 1372 Georgia Institute of Technology, Atlanta, Georgia 30332, United States; ‡ School of Chemical and Biomolecular Engineering, Georgia Institute of Technology, Atlanta, Georgia 30332, United States; § Wallace H. Coulter Department of Biomedical Engineering, Georgia Institute of Technology and Emory University, Atlanta, Georgia 30332, United States; ∥ School of Chemistry and Biochemistry, Georgia Institute of Technology, Atlanta, Georgia 30332, United States; ⊥ Department of Chemistry and Biochemistry, 8787 University of California Santa Cruz, Santa Cruz, California 95064, United States; # T-NeuroDx, Albuquerque, New Mexico 87123, United States; ¶ StemVax Therapeutics, a NovAccess Global Company, Chagrin Falls, Ohio 44023, United States; ∇ World Brain Mapping Foundation, Society for Brain Mapping & Therapeutics, Pacific Palisades, California 90272-3668, United States; ○ Parker H. Petit Institute for Bioengineering and Bioscience, Georgia Institute of Technology, Atlanta, Georgia 30332, United States

**Keywords:** amyloid beta, Alzheimer’s disease, protein deamidation, aggregation, inflammation, cytokines

## Abstract

The progressive aggregation of amyloid beta (Aβ) monomers into oligomers is a critical factor in Alzheimer’s disease (AD) pathogenesis. Although mutated forms of Aβ have been shown to display altered aggregation dynamics, the specific effects of deamidated Aβ on microglial function remain understudied. Our research group previously found that the deamidated variant Aβ-42-N27D modified Aβ aggregation, reduced neurotoxicity, and reduced microglial reactivity, but the impact of Aβ-42 side chain deamidation in general on such parameters remained unclear. Here, we expanded on our prior work by investigating how two site-specific Aβ-42 mutations (Q15E & N27D), where neutral amide side chains are replaced with negatively charged carboxylic acids, affect aggregation and microglial immune response using a mouse microglial cell line. Size exclusion chromatography, Thioflavin T fluorescence, circular dichroism, and dynamic light scattering revealed that Aβ-42-Q15E and Aβ-42-N27D exhibit reduced aggregation compared to Aβ-42 wild type (WT). Multiplexed analysis of 8 microglial cytokines secreted into the culture medium revealed that Aβ-42-Q15E and Aβ-42-N27D decrease the expression of inflammatory cytokines such as IL-6, IP-10, and MIP-1α relative to Aβ-42-WT. Immunocytochemistry revealed that Aβ-42-Q15E and Aβ-42-N27D decrease CD68 expression and intracellular Aβ labeling relative to Aβ-42-WT. These findings demonstrate that deamidation significantly alters Aβ-42 aggregation and microglial activation, suggesting structural modifications to Aβ-42 modulate inflammatory signaling in AD. This work provides a foundation for future studies on Aβ-42 post-translational modifications as potential therapeutic targets in AD.

## Graphical abstract

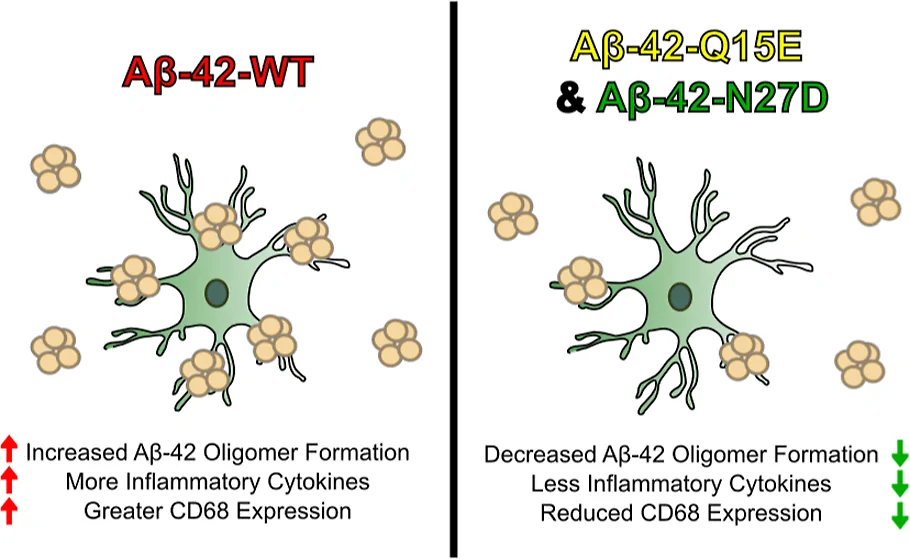

## Introduction

Alzheimer’s disease (AD) is a progressive neurodegenerative disorder that affects millions of people each year and is the leading cause of dementia globally.^1^ Accumulation of Aβ peptides into extracellular plaques is a defining hallmark of AD pathology.^2^ While it remains unclear whether Aβ plaques directly drive pathology, Aβ oligomers as well as chronic neuroinflammation have both been strongly implicated in AD pathogenesis.^3,4^ Microglia are the resident immune cells of the brain and function to clear cellular debris, prune synapses, and maintain homeostasis under healthy conditions.^5^ While microglia are believed to provide early protection during AD, microglial function becomes dysregulated during disease progression, reducing their ability to clear Aβ aggregates and contributing to a state of chronic neuroinflammation linked to cognitive decline.^6,7^

Given that Aβ aggregation state influences microglial activity,^8^ understanding how specific structural modifications to Aβ impact microglial activation is critical for elucidating mechanisms of inflammation in AD. In the healthy brain, microglia secrete neurotrophic factors that support neuronal health.^9^ However, in AD, oligomeric forms of Aβ deregulate signaling pathways vital for cellular function and gene expression.^10^ This dysregulation exacerbates chronic inflammation and accelerates neurodegeneration in AD patients.^11^ Aβ interacts with microglial surface receptors, such as TREM2, CD36, and toll-like receptors, resulting in dysregulated phospho-protein signaling cascades that drive expression of inflammatory cytokines, including IL-1β, IL-6, and TNF-α.^12^

In this study, we chose to focus on Aβ-42 as this form of Aβ is known to form more oligomers and be more neurotoxic than Aβ-40.^13^ Although limited, previous studies have explored how site-specific modification to Aβ variants influence aggregation and the resulting cellular responses.^14^ Importantly, deamidation at Q15 and N27 has been detected in vivo. Proteomic analysis of 5xFAD mouse and human brain samples has identified Aβ peptides carrying these modifications, confirming they occur in disease-relevant tissue.^15^ Our recent study examined Aβ-42 with a substitution at position 27, where asparagine was replaced with aspartic acid (Aβ-42-N27D) and found that this variant exhibited reduced aggregation compared to wild-type Aβ-42, and notably produced substantially lower quantities of soluble oligomers.^16^ In the current study, this result was successfully reproduced within a distinct laboratory environment, further validating the impact of Aβ-42-N27D on Aβ aggregation behavior. Our previous study also reported changes in microglial cytokine expression in response to the N27D variant. However, aspects of microglial activation and phagocytic function remained uncharacterized, providing the basis for our investigation into how deamidated Aβ-42 affects aggregation and broader microglial response.

Here, we investigated how site-specific deamidation of Aβ-42 influences its aggregation kinetics and microglial immune response. We found that Q15E and N27D mutants of Aβ-42 exhibited reduced aggregation, lower expression of inflammatory cytokines, and diminished CD68 expression compared to Aβ-42-WT. Our data indicate that Aβ-42 deamidation at either N27 or Q15 modifies its aggregation and immune activation, highlighting deamidation as a potentially general mechanism for attenuating Aβ-induced inflammation.

## Results

### Deamidation of Aβ-42 at Q15 and N27 Alters Aggregation

We began the current study by investigating how site-specific deamidation affects Aβ-42 aggregation. To do so, we used size exclusion chromatography (SEC) to monitor the aggregation profiles of Aβ-42-Q15E, Aβ-42-N27D, and Aβ-42-WT over 24 h. Freshly prepared monomers of each variant were isolated using SEC (Supporting Information Figure S1), diluted to 100 μM, and incubated at 37 °C for 24 h to allow aggregation to occur. The resulting aggregation profiles were then analyzed by reinjection onto the SEC column.

The SEC elution profiles revealed distinct aggregation behaviors among the three variants (Figures 1A and S2). Aβ-42-WT displayed a pronounced oligomeric peak at approximately 9 mL elution volume, indicating substantial formation of higher-order aggregates after 24 h of incubation. The deamidated variants, Aβ-42-N27D and Aβ-42-Q15E, showed reduced oligomeric peak formation, with peak heights approximately 75% lower than the wild-type variant. The monomeric peaks (eluting at ∼14–15 mL) remained prominent for both mutants, suggesting that deamidation reduces the progression from monomeric to oligomeric states.

**Figure 1:**
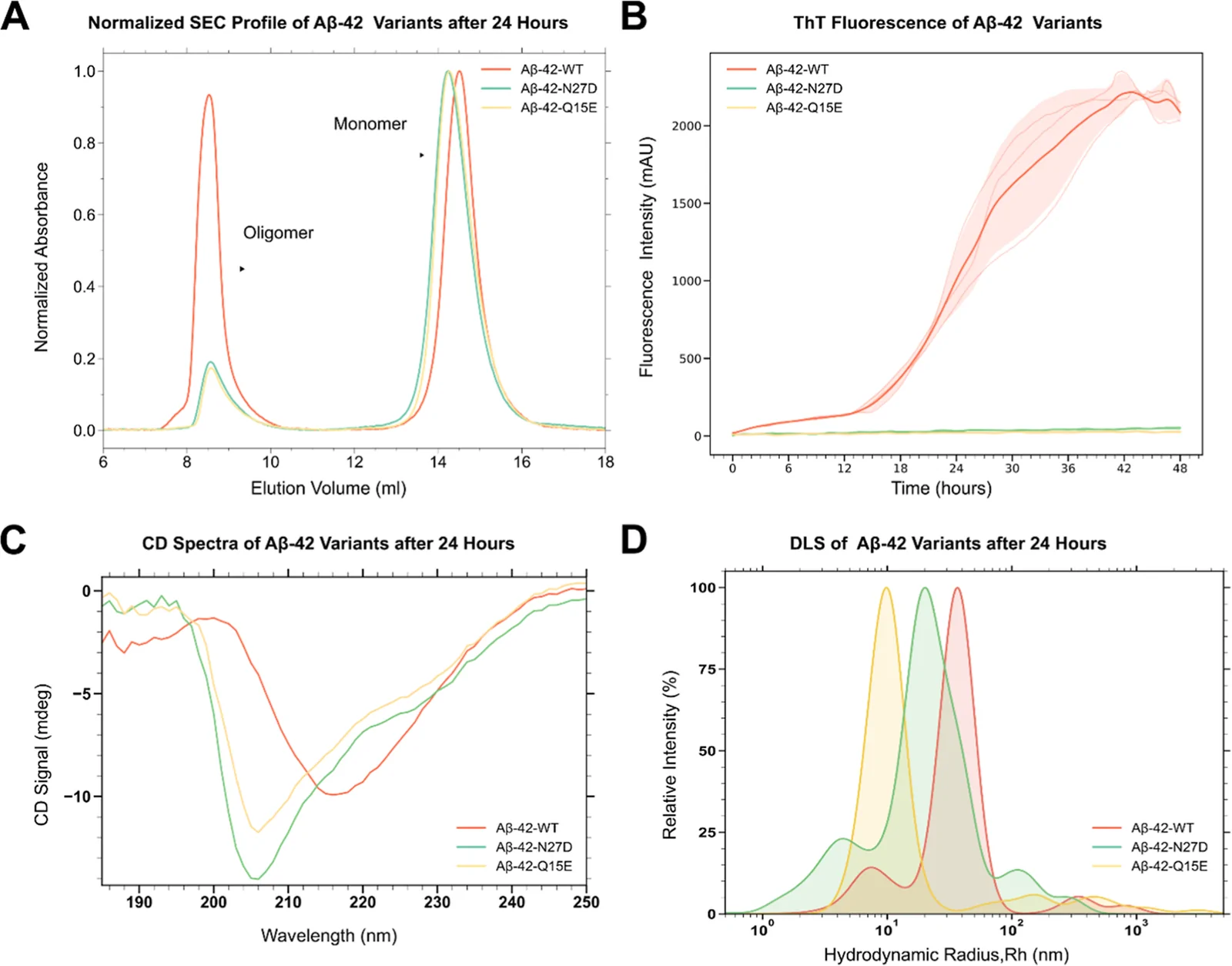
Deamidation of Aβ-42 at Q15 and N27 attenuates aggregation. (A) Aβ-42 monomers were collected using SEC (see Supporting Information Figure S1), diluted to 100 μM, and reinjected after 24 h incubation at 37 °C. SEC profiles showing difference in aggregates formed from incubated monomers of Aβ-42 WT, Aβ-42 N27D, and Aβ-42 Q15E after 24 h. (B) ThT fluorescence kinetics over 48 h (100 μM peptide, 20 mM NaPi pH 7.4, 37 °C, mean ± 1 SD of three replicate wells, *n* = 3). Aβ-42-WT shows the sigmoidal growth curve, reaching a plateau ThT fluorescence of 2171 ± 64 mAU. In contrast, both deamidation mimics remain near baseline (27 ± 4 and 49 ± 4 mAU for Q15E and N27D, respectively). (C) Far-UV CD spectra (250–185 nm, 25 °C; 1 mm path, 5 replicates averaged) of the same samples after 24 h. Aβ-42-WT shows the β-sheet band near 218 nm; Q15E and N27D retain a dominant ∼205 nm minimum consistent with more disordered, less β-sheet-like conformation than WT. (D) DLS cumulant Rh after 24 h (mean ± SD, *n* = 3), WT 29.5 ± 0.2 nm, Q15E 11.3 ± 0.8 nm, N27D 12.4 ± 4.9 nm.

To independently corroborate the SEC-based aggregation reported, we conducted three orthogonal biophysical readouts on the same SEC-isolated monomer preparations used for the aggregation analysis. All three measurements were performed on freshly SEC-isolated monomers of Aβ-42-Q15E, Aβ-42-N27D, and Aβ-42-WT at 100 μM in 20 mM sodium phosphate (NaPi, pH 7.4) and incubated at 37 °C for 24 h, matching the conditions used for SEC reinjection. Thioflavin T (ThT) fluorescence was used to quantify cross-β amyloid content as the variants aggregated, far-UV circular dichroism (CD) was used to track changes in secondary structure (Figures 1C and S3), and dynamic light scattering (DLS) was used to report the hydrodynamic size of the resulting species (Figure 1D). We additionally tested whether the formal net-charge difference between Aβ-42-WT (formal net charge −3 at pH 7.4) and the deamidation mimics (formal net charge −4) resolves at the bulk surface-potential level by ζ-potential measurement (Supporting Information Table S1). Together, these four readouts allow us to ask whether the reduced aggregation of the deamidation mimics is reproduced at the level of fibril content, secondary structure, and particle size and whether it tracks with a bulk electrostatic signature.

Thioflavin T fluorescence kinetics revealed a sharp variant-dependent difference in cross-β amyloid content. Aβ-42-WT exhibited the characteristic sigmoidal amyloid growth curve, reaching plateau ThT fluorescence of 2171 ± 64 mAU within the 48 h monitoring window. In contrast, both Aβ-42-Q15E and Aβ-42-N27D remained near baseline ThT signal across the full time course (plateau signals of 27 ± 4 and 49 ± 4 mAU, respectively), consistent with markedly attenuated fibril formation (Figure 1B). At the secondary-structure level, far-UV CD spectra acquired on each variant confirmed this difference. At *t* = 0 h, the freshly SEC-isolated monomers of all three variants gave essentially identical random-coil spectra (dominant negative band near 200 nm, no 218 nm minimum) (Supporting Information Figure S3). After 24 h of incubation, Aβ-42-WT developed the negative band centered near 218 nm diagnostic of β-sheet conformation, whereas both Aβ-42-Q15E and Aβ-42-N27D retained less-structured spectra lacking this minimum, with a dominant negative band near 205 nm (Figure 1C). DLS at the same 24 h time point independently corroborated the size phenotype: the cumulant hydrodynamic radius (Rh) of Aβ-42-WT was 29.5 ± 0.2 nm, whereas Aβ-42-Q15E (11.3 ± 0.8 nm) and Aβ-42-N27D (12.4 ± 4.9 nm) were ∼2.5-fold smaller, corresponding to a ∼15-fold reduction in particle volume (Figure 1D). Finally, ζ-potential measurements showed that the bulk surface potential of all three variants was submillivolt in magnitude and statistically indistinguishable (one-way ANOVA *F* = 4.93, *p* = 0.054; all pairwise Tukey HSD *p* > 0.05), and that the directional ordering did not match the formal net-charge prediction. Together, SEC, ThT, CD, and DLS converge on the same conclusion: Aβ-42-Q15E and Aβ-42-N27D form substantially less aggregated species than Aβ-42-WT under the conditions used here. The ζ-potential null result further indicates that this reduced aggregation does not arise from a bulk electrostatic repulsion mechanism.

### Deamidation of Aβ-42 Modulates Cytokine Expression in Mouse Microglial Cell Line

Chronic microglial activation is a hallmark of AD pathology and is closely linked to Aβ oligomerization.^17^ We therefore next investigated whether deamidated Aβ-42 variants would elicit distinct cytokine responses compared to Aβ-42-WT. To ensure comparability with prior mechanistic studies, we applied 50 nM of soluble Aβ (a physiologically relevant *in vitro* concentration^18,19^ ) of Aβ-42-WT, Aβ-42-N27D, and Aβ-42-Q15E to mouse microglial cells (MMCs) for 24 h. We then quantified 8 inflammatory cytokines associated with chronic inflammation secreted into the culture medium using a Luminex multiplexed immunoassay^20,21^ (Figure 2A).

**Figure 2:**
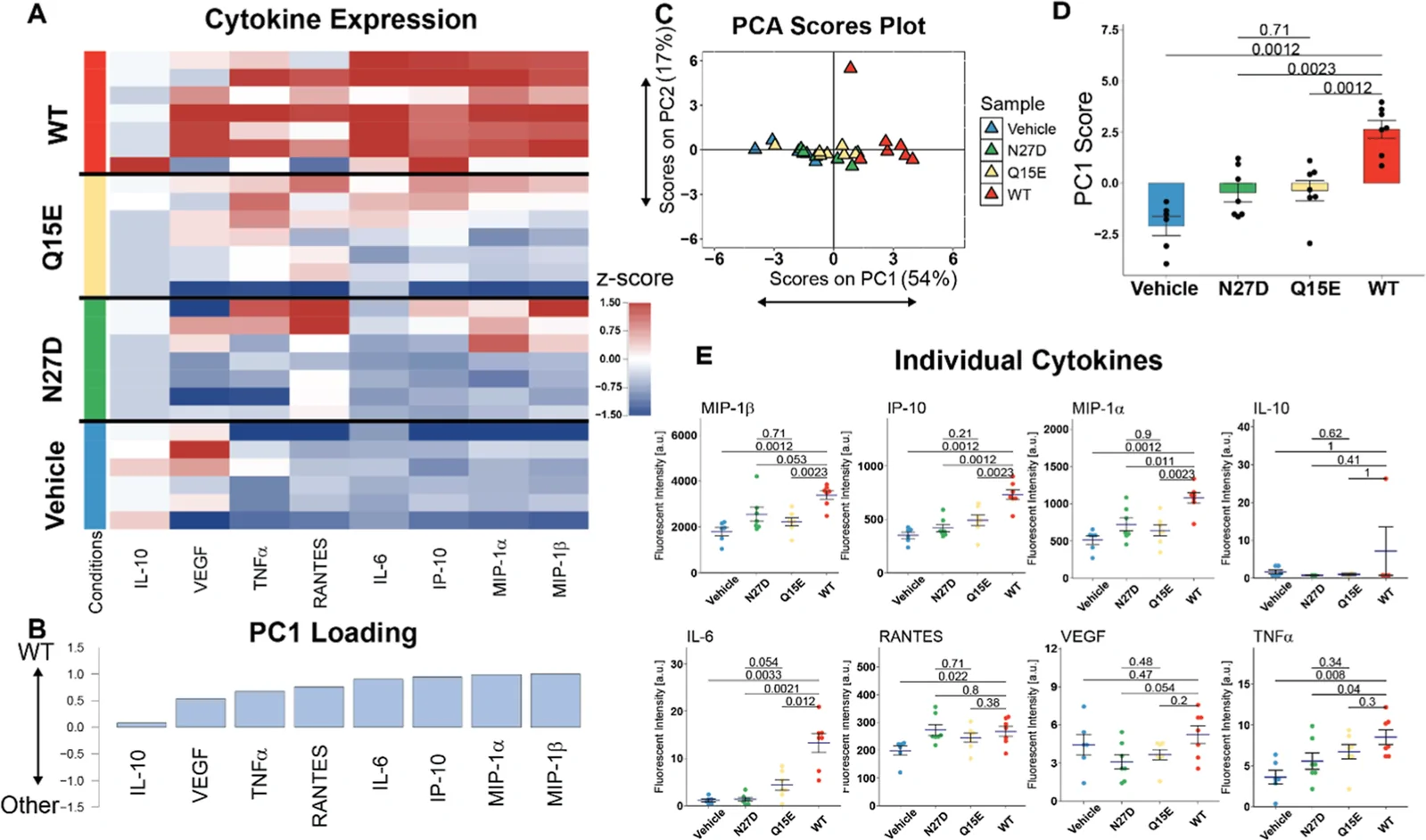
Deamidated Aβ modulates cytokine expression in mouse microglial cell line. (A) Multiplexed Luminex analysis of 8 cytokines (columns) expressed into the culture medium after 24 h of incubation with 50 nM of each Aβ variant (*n* = 6–8 wells/group). Each row in the *z*-scored heat map denotes an individual culture sample well. (B) PCA identified a variable, PC1, that captured the greatest axis of variation across experimental groups. PC1 consisted of a profile of cytokines that were upregulated with Aβ-42-WT. (C) Scores plot of principal component analysis (PCA). Each triangle represents a sample well. PC1 explained 54% of the variance, and PC2 explained an additional 17%. (D) Bar plot of PC1 scores for each group. In all bar plots, dots denote individual samples. (E) Dot plots for individual cytokines (mean ± SEM, Wilcoxon rank sum test). In all bar plots, dots denote individual samples. All *p*-values reflect Wilcoxon rank sum tests with Bonferroni adjustment for multiple comparisons. a.u., Arbitrary units.

Principal component analysis (PCA) identified principal components (PC1 and PC2) that captured the greatest variance in cytokine expression across samples. PC1 consisted of a cytokine profile and the loadings on PC1 reflected the relative influence of each cytokine. MIP-1β, MIP-1α, and IP-10 had the highest absolute loadings on PC1, indicating their most significant contribution to the PC1 score (Figure 2B). Scores on PC1 showed that Aβ-42-WT clustered separately on the far right, both Aβ-42-N27D and Aβ-42-Q15E clustered in the middle, while vehicle clustered on the left (Figure 2C). Similarly, PC1 scores were higher in Aβ-42-WT-treated cells compared to vehicle, Aβ-42-Q15E, and Aβ-42-N27D (Figure 2D). Thus, deamidation attenuates the PC1-associated cytokine response observed with Aβ-42-WT. MIP-1β, MIP-1α, IP-10, and IL-6 were significantly upregulated in Aβ-42-WT compared to vehicle, Aβ-42-N27D, and Aβ-42-Q15E (Figure 2E). Notably, Aβ-42-Q15E and Aβ-42-N27D elicited highly similar cytokine profiles, with only IL-6 showing a trend toward higher expression in response to Aβ-42-Q15E (Figure 2E). Together, both Aβ-42-N27D and Aβ-42-Q15E elicited lower cytokine expression compared to Aβ-42-WT, suggesting that Aβ deamidation may mitigate microglial dysregulation in AD.

### Aβ-42 Deamidation Attenuates CD68 Expression in Mouse Microglial Cell Line

Aβ-42 oligomers have been shown to increase microglial activation and dysregulate phagocytosis.^22^ CD68, a lysosomal marker, is consistently shown to be upregulated in activated microglia.^23^ Therefore, we utilized immunocytochemistry (ICC) to stain for CD68 in MMCs treated with 50 nM of Aβ-42-WT, Aβ-42-N27D, and Aβ-42-Q15E for 2 h, as this is a time period where studies have shown increased levels of Aβ-42 uptake.^24^ ICC examination of CD68 showed that CD68 labeling is reduced in vehicle, Aβ-42-N27D, and Aβ-42-Q15E (Figures 3A and S4). Quantification of CD68 fluorescent intensity normalized to DAPI cell count revealed reduced CD68 expression in Aβ-42-N27D and Aβ-42-Q15E compared to Aβ-42-WT (Figure 3B). CD68 levels did not differ between Aβ-42-N27D and Aβ-42-Q15E, indicating that Aβ deamidation reduces its potency to induce microglial activity.

**Figure 3:**
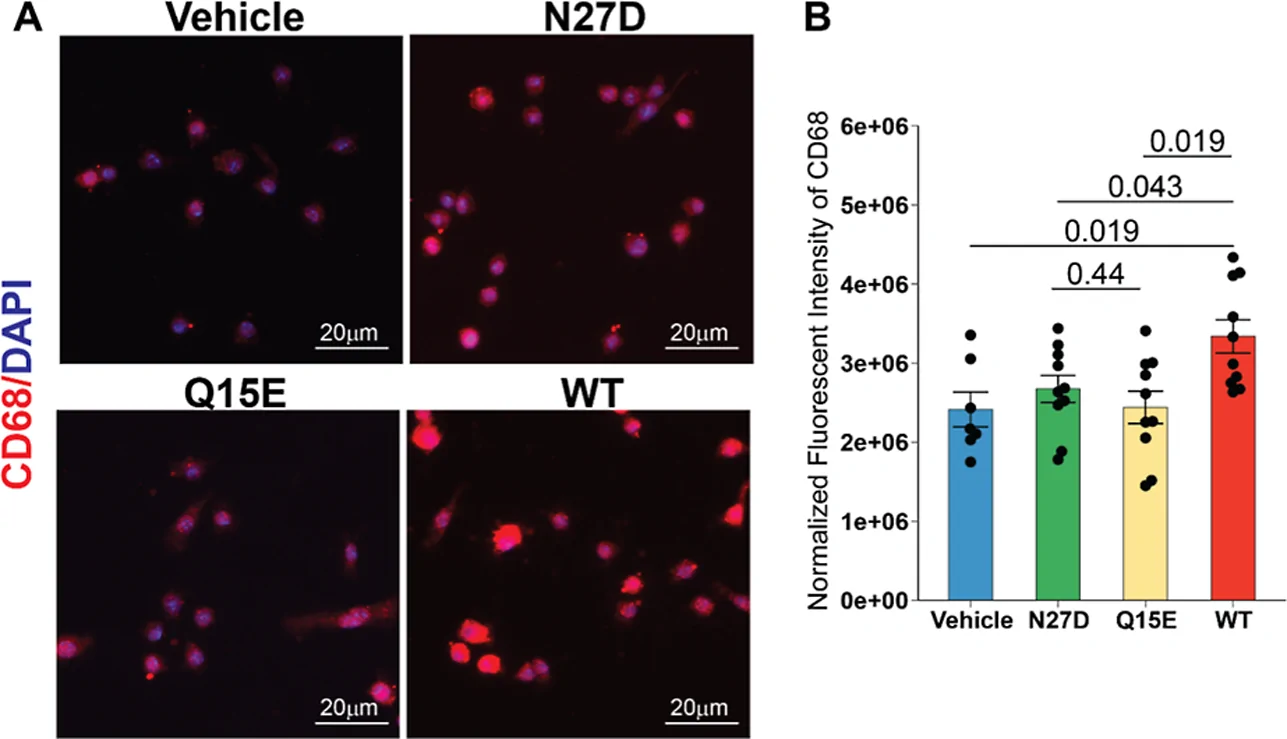
Deamidation abrogates Aβ-induced CD68 expression in mouse microglial cell line. (A) ICC for CD68 (red) and DAPI (blue) after 2 h of incubation with 50 nM of each Aβ variant (scale bar: 20 μm, representative sections from *n* = 12–13 sample well/group). (B) Integrated CD68 fluorescent intensity normalized by number of nuclei (*n* = 12–13, mean ± SEM, Wilcoxon rank sum tests with Bonferroni adjustment for multiple comparisons).

### Deamidation Modulates Aβ-42 Internalization in Mouse Microglial Cell Line

Given that Aβ-42-Q15E and Aβ-42-N27D elicited lower CD68 expression than Aβ-42-WT, we next asked whether these differences reflected altered peptide internalization. To assess this, we performed ICC for 6E10, an antibody that recognizes an epitope within residues 4–10 of the Aβ peptide.^25^ MMCs were treated with 50 nM of Aβ-42-Q15E, Aβ-42-N27D, Aβ-42-WT, or vehicle for 2 h, matching the time point used for CD68 measurements. Visual inspection revealed reduced 6E10 labeling in cells treated with Aβ-42-Q15E and Aβ-42-N27D relative to Aβ-42-WT (Figures 4A and S5). Quantification of integrated 6E10 fluorescence normalized to DAPI cell count revealed significantly reduced labeling in Aβ-42-Q15E compared to Aβ-42-WT, whereas Aβ-42-N27D exhibited a similar downward trend (Figure 4B). Direct comparison of the two deamidated variants revealed that 6E10 labeling trended lower in Aβ-42-Q15E relative to Aβ-42-N27D, although this difference did not reach statistical significance. Together, these data indicate that deamidation reduces peptide internalization relative to Aβ-42-WT, with Aβ-42-Q15E reaching statistical significance and Aβ-42-N27D showing a consistent but nonsignificant trend.

**Figure 4:**
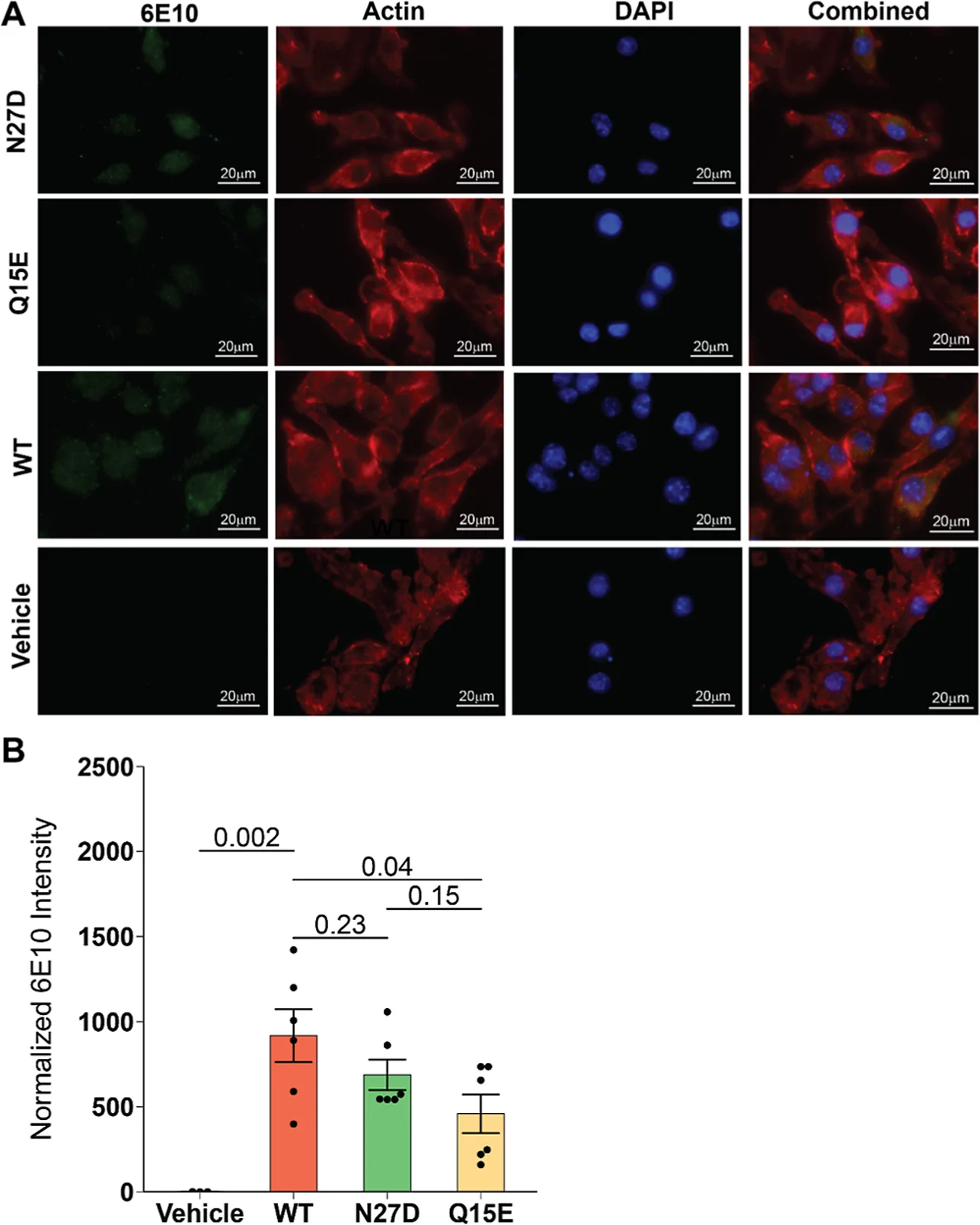
Deamidation of Aβ-42 results in diminished internalization at 2 h post treatment. (A) ICC for 6E10 (green), F-actin (red), and DAPI (blue) after 2 h of incubation with 50 nM of each Aβ variant (scale bar: 20 μm, representative sections from *n* = 6 sample well/group). (B) Integrated 6E10 fluorescent intensity normalized by number of nuclei (*n* = 6, mean ± SEM, Wilcoxon rank sum tests with Bonferroni adjustment for multiple comparisons).

## Discussion

Aβ oligomers are widely recognized as drivers of microglial activation, inflammation, and neurodegeneration.^17,26^ Thus, Aβ mutants that promote oligomerization would be expected to induce stronger microglial activation than mutants that favor monomeric forms. Building on our previous study where we examined the impact of Aβ-42-N27D on aggregation and microglial activity,^16^ this work differs methodologically in that we freshly prepared our samples by isolating monomers using SEC to remove any preseeding effects that could affect the aggregation kinetics and behavior of the Aβ-42 peptide. This modification led to a more consistent microglial response than observed previously.

Our SEC data indicate that Aβ-42-Q15E and Aβ-42-N27D aggregate less than Aβ-42-WT. Therefore, the observed differences in microglial activity could be partially due to altered oligomer formation. Similar findings have been reported for Aβ-40, where the Aβ-40-Q15E and Aβ-40-N27D variants showed slower aggregation compared to Aβ-40-WT.^14^ Thus, if microglial activation were solely dependent on aggregation state, these findings would support the conclusion that Aβ-42-Q15E and Aβ-42-N27D are less activating than Aβ-42-WT. ThT fluorescence remained near baseline for both mimics over 48 h while Aβ-42-WT followed the characteristic sigmoidal growth curve;^14,27^ CD spectra showed random-coil character (negative band near 200 nm, no 218 nm minimum) for the monomers at *t* = 0 h. After 24 h, Aβ-42-Q15E and Aβ-42-N27D retained a substantially more disordered signature (minimum ∼205 nm), while Aβ-42-WT developed the diagnostic β-sheet signature (minimum ∼216 nm);^28^ DLS showed ∼2.5-fold smaller hydrodynamic radii and a ∼15-fold reduction in particle volume for both mimics. Convergence of four independent methods (SEC, ThT, CD, DLS) argues that this is a robust phenotype rather than a method-specific artifact. Q15E and N27D suppressed oligomer formation, ThT fluorescence, β-sheet content, and assembly size to nearly identical extents, despite occupying structurally distinct regions of Aβ-42: Q15E in the central hydrophobic core and N27D in the turn region. This convergence points to a shared property of the two substitutions, namely the addition of a negative charge and the loss of a side-chain amide hydrogen-bond donor, rather than position-specific structural disruption.

To test whether the formal charge difference between WT and the deamidation variants manifests as a bulk electrostatic effect, we measured ζ-potential under low-salt conditions (10 mM NaPi, κ^–1^ ≈ 1.77 nm) chosen to maximize sensitivity (Supporting Information Table S1). |ζ| was submillivolt for all three variants, pairwise differences did not reach significance (one-way ANOVA *F* = 4.93, *p* = 0.054), and the directional ordering did not match the prediction that the tetra-anionic Q15E and N27D would exhibit larger |ζ| than trianionic WT. The likely interpretation is counterion condensation onto Aβ-42’s dense N-terminal acidic cluster,^29^ which screens the additional carboxylate before it reaches the slip plane. We therefore attribute the reduced aggregation propensity to local side-chain effects at the substituted position such as the desolvation penalty of the introduced carboxylate combined with loss of the parental Asn/Gln amide hydrogen-bond donor within the cross-β nucleus^30,31^ rather than bulk electrostatic repulsion.

While the aggregation dynamics of deamidated mutants of Aβ-40 have been documented,^14^ the aggregation dynamics and microglial response to Aβ-42-Q15E and Aβ-42-N27D remain poorly understood. Across SEC, ThT, CD, and DLS, Aβ-42-Q15E and Aβ-42-N27D aggregate similarly to one another and substantially less than Aβ-42-WT, despite the two substitutions occupying structurally distinct regions of the peptide. Research has shown that the loss of negative charges at specific residues on Aβ-42 leads to increased aggregation, enhanced stability, and greater neurotoxicity.^32^ Conversely, both Q15E and N27D introduce a negative charge, consistent with the slower aggregation we observe. However, our ζ-potential data indicate that this effect does not manifest as a bulk electrostatic difference and is more plausibly explained by local side-chain effects at the substituted position.^33,34^

One major finding in our study is that both Aβ-42-Q15E and Aβ-42-N27D reduce microglial activation and diminish expression of inflammatory cytokines compared to Aβ-42-WT. As Aβ-42-WT forms more oligomers than Aβ-42-Q15E and Aβ-42-N27D, Aβ-42-WT could be able to bind to more microglial surface receptors than the deamidated mutants. Microglia express receptors such as TREM2 that preferentially recognize oligomeric Aβ species.^35,36^ TREM2 signaling is closely linked to microglial activation and inflammatory cytokine production, providing a mechanism for how differences in oligomer abundance may translate into altered inflammatory responses.^35^ The reduced oligomer populations in Aβ-42-Q15E and Aβ-42-N27D could explain the decreased inflammatory cytokine expression observed in the deamidated mutants.^37,38^ While limited differences were found between Aβ-42-Q15E and Aβ-42-N27D in terms of cytokine expression, our data suggest that Aβ-42-Q15E and Aβ-42-N27D may interact differently with microglia.^39^ One such analyte, IL-6, trended higher in microglia treated with Aβ-42-Q15E than Aβ-42-N27D (*p* = 0.054). Since IL-6 production is regulated by multiple receptor-mediated signaling pathways,^40,41^ these findings suggest that Aβ-42-Q15E and Aβ-42-N27D may engage distinct microglial surface receptors. This suggestion is plausible given that the deamidation sites differ between the two mutants, potentially altering their conformation and resultant receptor binding.

In addition to cytokine profiling, we quantified CD68 because it is upregulated in AD pathology and cognitive decline.^23^ We found that both Aβ-42-Q15E and Aβ-42-N27D induce significantly lower expression of CD68 compared to Aβ-42-WT. Since CD68 is a marker of lysosomal activity, it also serves as an indirect measure for evaluating lysosomal degradation of Aβ-42.^23^ To further interrogate this, we performed ICC for 6E10 at the same 2 h time point and found reduced intracellular Aβ labeling in the deamidated variants relative to Aβ-42-WT. This could reflect either reduced peptide internalization or more efficient lysosomal processing of the deamidated variants at this early time point, both of which would be consistent with the lower CD68 expression observed. Collectively, the convergence of attenuated cytokine expression, reduced CD68, and lower intracellular Aβ labeling suggests that deamidation at Q15 or N27 may alter how microglia engage with Aβ-42, whether by reducing the inflammatory burden of internalized peptide, enhancing its clearance efficiency, or both.

The N27D and Q15E mutations were designed to mimic spontaneous deamidation events that disrupt aggregation. Both asparagine and glutamine residues contribute to fibril stability through interdigitated hydrogen bonding networks observed in fibril structures (PDB 5OQV and 2NAO).^30,42^ The N27D mutation substitutes asparagine with aspartic acid at residue 27 within the turn region (residues 22–29), a structurally sensitive area important for β-hairpin formation during aggregation and oligomerization.^30,42–45^ Similarly, Q15E introduces a negative charge within the central hydrophobic core, where Q15 normally contributes to fibril stability.^14,30^ The introduction of negatively charged residues at these positions likely disrupts aggregation through local side-chain effects, including loss of stabilizing hydrogen bonding interactions at the substituted position, consistent with previous observations that charge manipulations at specific residues modulate Aβ aggregation propensity and neurotoxicity.^46^ However, detailed structural characterization will be necessary to fully elucidate the mechanisms by which these deamidation-mimicking mutations alter Aβ-42 aggregation behavior.

While our study provides important insights into how deamidated Aβ-42 mutants modulate microglial activation, several limitations should be acknowledged. First, the experiments were conducted in an immortalized microglial monoculture, which shares key characteristics with primary microglia but does not fully capture the complexity of primary microglial phenotypes,^47–49^ particularly in the context of AD. It is also worth noting that while 50 nM was selected as a physiologically relevant Aβ concentration to enable controlled comparison across variants, local concentrations in synaptic compartments may transiently exceed this level due to geometric constraints.^50^ Therefore, future studies could interrogate cellular responses across a range of Aβ concentrations to better capture the diversity of microglial environments in vivo. Additionally, although CD68 staining modulation suggests deamidation altered phagocytic activity, CD68 primarily reflects lysosomal activity and does not directly measure microglial Aβ uptake via phagocytosis, a process heavily implicated in AD pathology.^51^ Also, our cytokine profile suggests that the two deamidated Aβ-42 variants may elicit slightly different microglial responses, motivating future analysis of receptor binding kinetics or downstream signaling activation. Together, these limitations highlight the need for in vivo studies and expanded mechanistic analyses.

Overall, our data show that deamidation of Aβ-42 reduces aggregation and dampens microglial activation. Given the growing clinical significance of Aβ aggregation and immune activity in AD,^52^ our findings therefore support further interrogation of Aβ-42 deamidation as a clinically relevant approach to reducing its toxicity. In particular, the formulation of new pharmacological strategies for post-translational deamidation could represent a viable strategy for AD treatment.

## Methods

### Aβ Preparation

Aβ-42-WT, Aβ-42-N27D, and Aβ-42-Q15E peptides were synthesized using a CEM Liberty Blue peptide synthesizer in-house using Fmoc solid-phase peptide synthesis following our previously established protocol.^53–55^ Peptides were verified for molecular weight using electrospray ionization mass spectrometry (ESI-MS) at the proteomics facility, confirming the presence of peptides with correct molecular masses corresponding to their expected sequences. See Supporting Information Figure S6.

### Monomer Isolation

For monomer isolation, lyophilized peptides were dissolved in 50 mM sodium hydroxide (NaOH) and allowed to equilibrate for 20 min at room temperature to ensure complete dissolution. The peptide solutions were then immediately subjected to size exclusion chromatography (SEC) to isolate monomeric fractions.

### Size Exclusion Chromatography

Size exclusion chromatography (SEC) was performed using a Superdex 75 increase 10/300 GL column (GE Healthcare) connected to an ÄKTA GO chromatography system. The running buffer consisted of 20 mM sodium phosphate (NaPi, pH 7.4). Peptide samples (3 mL) were injected at a flow rate of 0.5 mL/min, and elution was monitored by UV absorbance at 280 nm. Monomeric fractions were identified based on their elution volume (∼14 mL) and collected accordingly. The isolated monomers were immediately diluted to a final concentration of 100 μM in the same buffer and used for aggregation studies.

For aggregation analysis, freshly isolated monomers were incubated at 37 °C for 24 h without agitation. Following incubation, samples were reinjected into the SEC column under identical conditions to analyze the aggregation state.

### Thioflavin T Fluorescence Assay

Thioflavin T (ThT) fluorescence kinetics were acquired on a BioTek Synergy H1 in 96-well, black-walled, clear bottom microplates. Freshly SEC-isolated Aβ-42-WT, Aβ-42-Q15E, and Aβ-42-N27D monomers were prepared in 20 mM NaPi pH 7.4 at a final peptide concentration of 100 μM and added to wells containing ThT to give a final ThT concentration of 20 μM in the well. Plates were sealed with optical-clear film and incubated at 37 °C, and readings were taken at 15 min intervals over 48 h. Three replicate wells were collected per variant in each independent experiment, and the per-well kinetic traces were buffer-subtracted, normalized, and averaged within variants prior to plotting.

### Circular Dichroism Spectroscopy

Far-ultraviolet circular dichroism (CD) spectroscopy was performed on an AP Chirascan-plus CD spectrometer using a 1 mm path-length quartz cuvette. Freshly SEC-isolated Aβ-42-WT, Aβ-42-Q15E, and Aβ-42-N27D monomers were prepared at 100 μM in 20 mM NaPi pH 7.4. Spectra were recorded on the freshly isolated monomers (*t* = 0 h) and again after incubation at 37 °C for 24 h, matching the conditions used for SEC reinjection, ThT, and DLS. Spectra were recorded from 250 to 185 nm, with five accumulations averaged per spectrum and a matched-buffer blank subtracted from each variant spectrum prior to plotting.

### Dynamic Light Scattering

Dynamic light scattering (DLS) measurements were performed on DynaPro multimodal DLS instrument. Freshly SEC-isolated Aβ-42-WT, Aβ-42-Q15E, and Aβ-42-N27D monomers were prepared at 100 μM in 20 mM NaPi pH 7.4 and incubated at 37 °C for 24 h prior to acquisition, matching the conditions used for SEC reinjection and ThT. Three wells per variant were loaded into the microcuvette plate, and ten 10 s acquisitions were collected per well at 25 °C. Cumulants analysis was performed in the instrument software with the default aqueous-buffer viscosity correction to report the hydrodynamic radius (Rh) for each well. Per-well Rh values were averaged within variant to report a mean ± SD across the *n* = 3 replicate wells.

### Zeta-Potential Measurements

ζ-Potential measurements were performed on a Malvern Zetasizer Nano ZS using DTS1070 disposable folded capillary cells. Freshly SEC-isolated Aβ-42-WT, Aβ-42-Q15E, and Aβ-42-N27D monomers were prepared at 100 μM in 20 mM NaPi pH 7.4 (matching the conditions used for SEC, ThT, CD, and DLS) and then 2-fold diluted with Milli-Q water immediately prior to injection into the capillary cell, giving a final measurement composition of 50 μM peptide in 10 mM NaPi. The 2× dilution step was introduced because initial ζ-potential acquisitions on undiluted samples (100 μM, 20 mM NaPi) produced substantial sample foaming and rapid Joule-heating-induced aggregation during the applied-voltage measurement (consistent with the higher solution conductivity and corresponding current density at 20 mM ionic strength), which compromised measurement reproducibility. At 10 mM NaPi, the Debye screening length is extended to κ^–1^ ≈ 1.77 nm (versus ∼1.25 nm at 20 mM), increasing sensitivity to any charge-dependent surface-potential difference between variants. The Smoluchowski approximation was used to convert electrophoretic mobility to ζ-potential. Three independent technical replicates were collected per variant. Group-level differences were assessed by one-way ANOVA (*F* and *p* reported in the table caption) followed by Tukey HSD posthoc comparisons for the three pairwise contrasts.

### Microglial Cell Line

The MMCs used in this study were generated by immortalizing primary C57BL/6 microglia using the J2 retrovirus.^56^

### Cell Conditioning and Media Collection

Mouse microglial cells (MMCs) were cultured in DMEM-F12 medium supplemented with 10% fetal bovine serum and 1% penicillin–streptomycin until they reached 70% confluency. Cells were stored in an incubator with 95% air and 5% CO_2_ atm at 37 °C. Upon reaching 70% confluency, cells were seeded into 12-well plates and incubated for 24 h. Cells were treated with either 50 nM Aβ-42-WT, 50 nM Aβ-42-N27D, 50 nM Aβ-42-Q15E or vehicle (20 mM NaPi) for 24 h followed by medium collection. Collected medium was centrifuged at 13,000 rpm for 10 min at 4 °C, then the supernatant was collected and stored at −80 °C. Multiplexed cytokine quantification was conducted using the Milliplex MAP Mouse Cytokine/Chemokine 18-Plex kit (Eotaxin*, GM-CSF*, IFN-γ*, IL-1β*, IL-2*, IL-3*, IL-4*, IL-6, IL-10, IL-12p70*, IL-13*, IP-10, M-CSF*, MIP-1α, MIP-1β, RANTES, TNFα, and VEGF) (Millipore Sigma MCYTOMAG-70K). Cytokines marked with an asterisk did not fall within a linear range of bead fluorescent intensity vs protein concentration and were not included in the analysis.

### Multivariate Analysis of Cytokine Data

Data were analyzed and figures were generated via RStudio (Boston, MA) using the R programming language. The tidyverse collection of packages was used to conduct data processing. R package heatmap3 was used to generate heatmaps; R packages ggplot2, ggpubr, ggbeeswarm, and ggprism were used to generate dotplots and barplots. Clustering was conducted using the hclust function of the stats package in R using Euclidean distance in the unweighted pair group method with arithmetic mean. For functional and protein analysis, Bonferroni adjustment was applied to correct for multiple comparisons.

Multivariate cytokine data were analyzed using principal component analysis (PCA) to identify axes, called principal components (PCs), that capture the greatest variance in cytokine expression across samples. PCA was performed using the R package ropls, with all data *z*-scored prior to analysis. Two principal components were extracted, and 5-fold cross-validation was applied to evaluate model robustness. All cytokine data were *z*-scored prior to analysis. PCA loadings were used to represent the contribution of each analyte to the observed variance.

### Cell Conditioning for CD68 and 6E10 Analysis

Mouse microglial cells were cultured in DMEM-F12 medium supplemented with 10% fetal bovine serum and 1% penicillin–streptomycin until they reached 70% confluency. Cells were stored in an incubator with 95% air and 5% CO_2_ atm at 37 °C. Upon reaching 70% confluency, cells were seeded into a 96-well plate and incubated for 24 h. Cells were treated with either 50 nM Aβ-42-WT, 50 nM Aβ-42-N27D, 50 nM Aβ-42-Q15E or vehicle (20 mM NaPi) for 2 h followed by fixing with 4% PFA on ice for 10 min. Cells were then rinsed 3× with cold PBS and then stored at 4 °C.

### Immunocytochemistry

Fixed cells were permeabilized with 0.01% Triton X-100 in PBS for 10 min at room temperature, then rinsed twice with PBS. Cells were blocked for 1 h at room temperature using blocking buffer containing 5% BSA and 3% goat serum in PBS. Following blocking, cells were incubated overnight at 4 °C with rat anti-CD68 antibody (1:200; ThermoFisher) or mouse anti-β-amyloid 1–16 (6E10) (1:500; BioLegend). The following day, cells were washed five times with PBS containing 5% BSA, with each wash lasting 5 min. Wells were then incubated with the appropriate secondary antibody for 2 h at room temperature: Alexa Fluor 555 conjugated secondary antibody (1:2000; ThermoFisher) for CD68, or Alexa Fluor 488 conjugated secondary antibody (1:2000; ThermoFisher) for 6E10. Afterward, cells were washed three times with 5% BSA in PBS for 5 min each wash. Nuclei were counterstained with DAPI for 20 min at room temperature, and cells stained for 6E10 were counterstained with rhodamine phalloidin (F-actin) (1:100; ThermoFisher), followed by three additional 5 min washes with 5% BSA in PBS. Cells were then rinsed three times with PBS and stored in PBS until imaging. Samples were imaged using epifluorescent microscopy on a Zeiss Axio Observer Z.1 inverted microscope with a 20× lens and halogen bulb illumination using Zeiss filter set 49 to image DAPI, Zeiss filter set 38 to image Alexa Fluor 488, and Zeiss filter set 20 to image Alexa Fluor 555. Quantification of CD68/6E10 fluorescent intensity was analyzed by dividing the CD68/6E10 intensity by DAPI cell counts (ImageJ Macro). See Supporting Information Table S2 for antibody catalog numbers.

### ImageJ Macro

An ImageJ macro was developed to automate the quantification of CD68 fluorescence intensity. The macro recursively scans a directory for merged images containing targeted red (CD68), green (6E10), and blue (DAPI) channels, while excluding single-channel files. Each image was then split into individual channels. The DAPI channel is thresholded using the Otsu method and segmented via watershed to count DAPI-positive nuclei, while measuring the red/green channels’ integrated fluorescence intensity. The intensity value was then normalized to the number of DAPI-positive nuclei to account for cell density. Finally, output values were printed to a log for export.

## Supplementary Material



## Data Availability

The data described in the manuscript are located within the manuscript and Supporting Information.

## Notes

Bulk ζ-potential of all three Aβ-42 variants, table of antibody information, monomer isolation via SEC, non-normalized SEC profiles of Aβ-42 variants, far-UV CD spectra at *t* = 0 h, representative images of CD68 staining, representative images of 6E10 staining, ESI-MS verification of synthesized Aβ-42 peptides (PDF)
