# Supplementary material for: Aβ-42 Side Chain Deamidation at Q15 or N27 Modulates Protein Aggregation and Alters Microglial Cytokines and CD68
Source: ACS Chem Neurosci. 2026 Sep 8;17(20):3421–30. doi: 10.1021/acschemneuro.6c00024 (PMC13693379; doi:10.1021/acschemneuro.6c00024)
Supplement: Supplementary file 1 [file cn6c00024_si_001.pdf]

Aβ-42 sidechain deamidation at Q15 or N27 modulates protein aggregation and alters microglial cytokines and CD68

Martin N. Griffin[1+], Daniel M. Dinakarapandian[2+], Chenxing Li[3], Pranay Ramaswamy[4], Sia J. Saheba[3], Janie Lee[3], Tarunya Rao Sudarshan[2], Maria Sajimon[5], Christopher J. Wheeler[5,6,7,8], Jevgenij A. Raskatov[5], Anant K. Paravastu[2,9*], Levi B. Wood[1,3,9*]

[1] George W. Woodruff School of Mechanical Engineering, Georgia Institute of Technology, Atlanta, GA 30332, USA
[2] School of Chemical and Biomolecular Engineering, Georgia Institute of Technology, Atlanta, GA 30332, USA
[3] Wallace H. Coulter Department of Biomedical Engineering, Georgia Institute of Technology and Emory University, Atlanta, GA 30332, USA
[4] School of Chemistry and Biochemistry, Georgia Institute of Technology, Atlanta, GA 30332, USA
[5] Department of Chemistry and Biochemistry, University of California Santa Cruz, Santa Cruz, CA 95064, USA
[6] T-NeuroDx, Albuquerque, NM 87123, USA
[7] StemVax Therapeutics, a NovAccess Global Company, Chagrin Falls, OH 44023, USA
[8] World Brain Mapping Foundation, Society for Brain Mapping & Therapeutics, Pacific Palisades, CA 90272-3668, USA
[9] Parker H. Petit Institute for Bioengineering and Bioscience, Georgia Institute of Technology, Atlanta, GA 30332, USA

+ Equally contributing authors

* Corresponding Authors

Supporting Information

Supporting Information Tables

Supporting Table 1. Bulk ζ-potential of Aβ-42 variants measured by phase-analysis light scattering (Malvern Zetasizer, M3-PALS) at 25 °C.

Variant	Formal net charge at pH 7.4	ζ-potential, mean ± SD (mV)	n
**Aβ-42-WT**	−3	−0.94 ± 0.41	3
**Aβ-42-N27D**	−4	−0.11 ± 0.44	3
**Aβ-42-Q15E**	−4	−0.22 ± 0.10	3

Sample composition: 50 µM peptide in 10 mM sodium phosphate (pH 7.4), obtained by 2-fold MilliQ-water dilution of a 100 µM stock in 20 mM NaPi (the latter matching the SEC, ThT, CD, and DLS conditions used elsewhere in this study). Values are mean ± standard deviation of $n = 3$ technical replicates per variant. One-way ANOVA across the three variants: $F = 4.93$, $p = 0.054$. Pairwise Tukey HSD post-hoc comparisons: WT vs N27D $p = 0.063$; WT vs Q15E $p = 0.10$; N27D

vs Q15E $p = 0.93$. All three variants exhibit $|\zeta| < 1.5$ mV with no statistically significant pairwise differences; the magnitude ordering does not match the prediction that the formally tetra-anionic deamidation mimics (Q15E, N27D) should exhibit larger $|\zeta|$ than the formally tri-anionic WT.

Supporting Table 2. Antibody Information

Antibody	Supplier	Catalog #	Dilution
CD68	ThermoFisher	14-0681-82	1:200
6E10	BioLegend	803001	1:500
F-Actin	ThermoFisher	R415	1:100

Supporting Information Figures

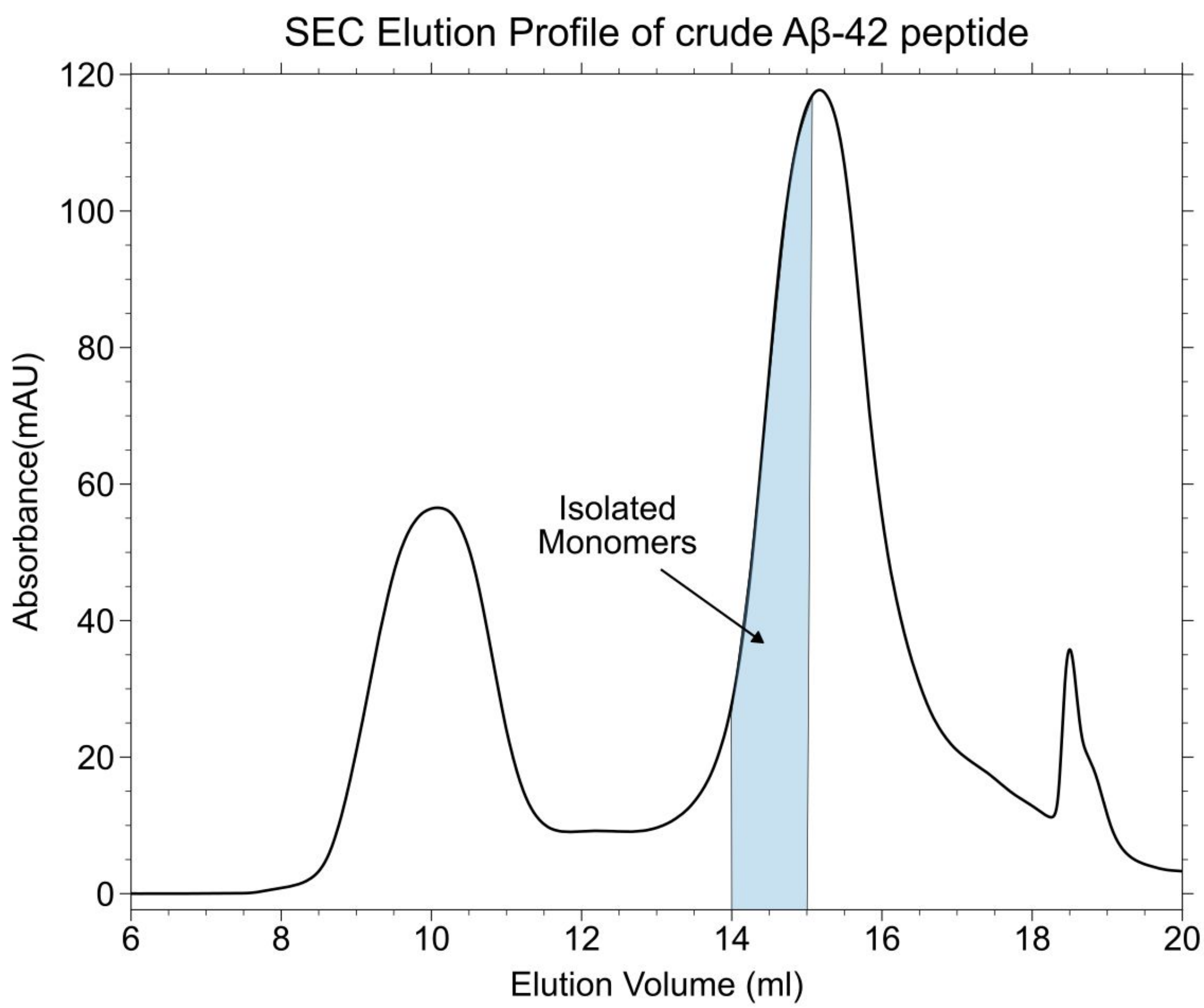

Supporting Figure 1. Aβ-42 monomers isolation using Size Exclusion Chromatography. Crude Aβ-42 peptides were dissolved in 50 mM NaOH and injected into SEC column at 0.5 mL/min flow rate. The monomers eluted at 14-15 mL (highlighted area in blue) were collected and diluted to the final concentration of 100 µM. The collected monomers were incubated at 37 °C for 24 hours.

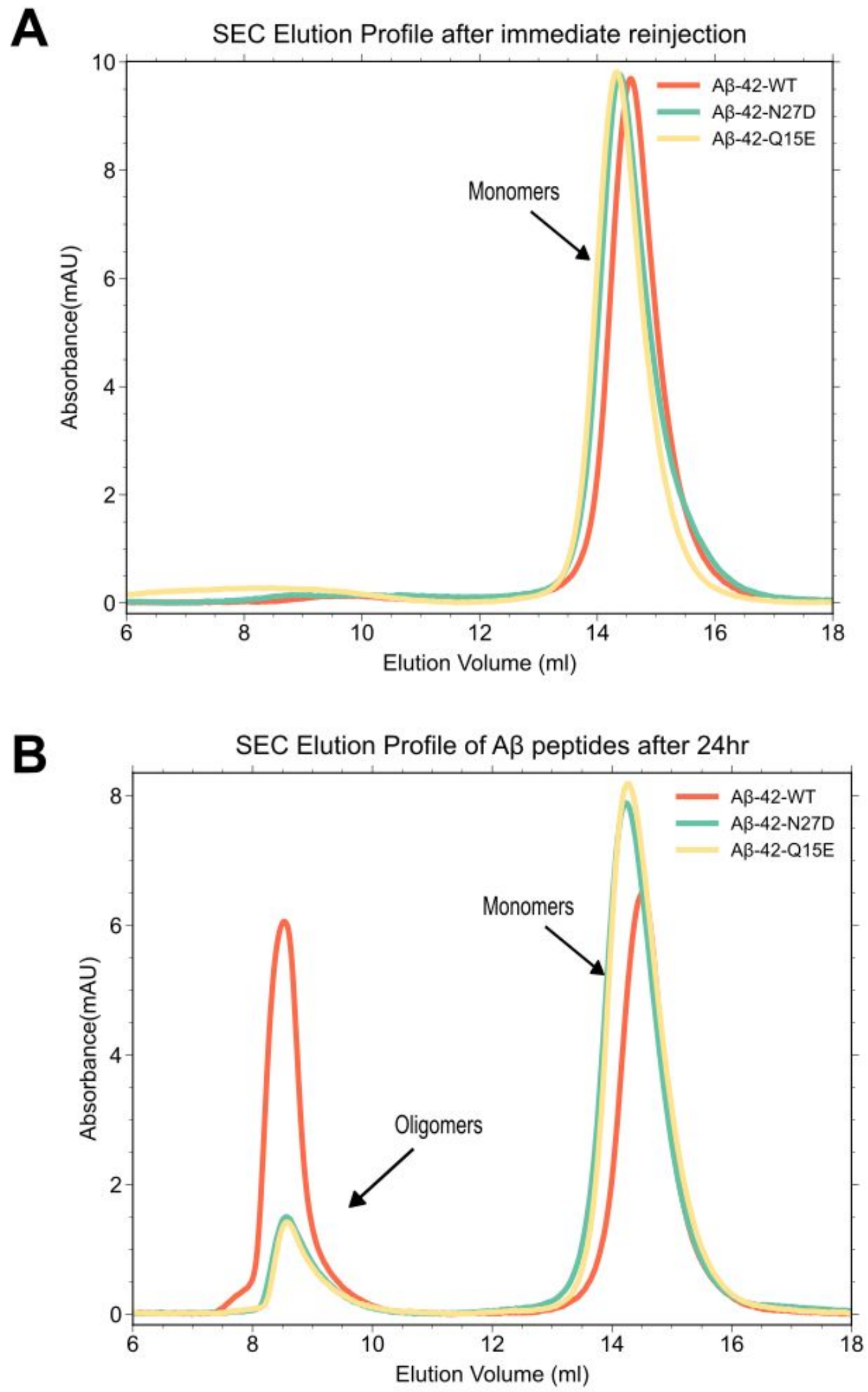

Supporting Figure 2. Non-normalized SEC profiles of Aβ-42 peptides. A. Immediate reinjection of freshly isolated monomers at t = 0 hr. **B.** SEC profiles of incubated monomers of Aβ-42-WT, Aβ-42-Q15E, and Aβ-42-N27D after 24 hours at 37 °C.

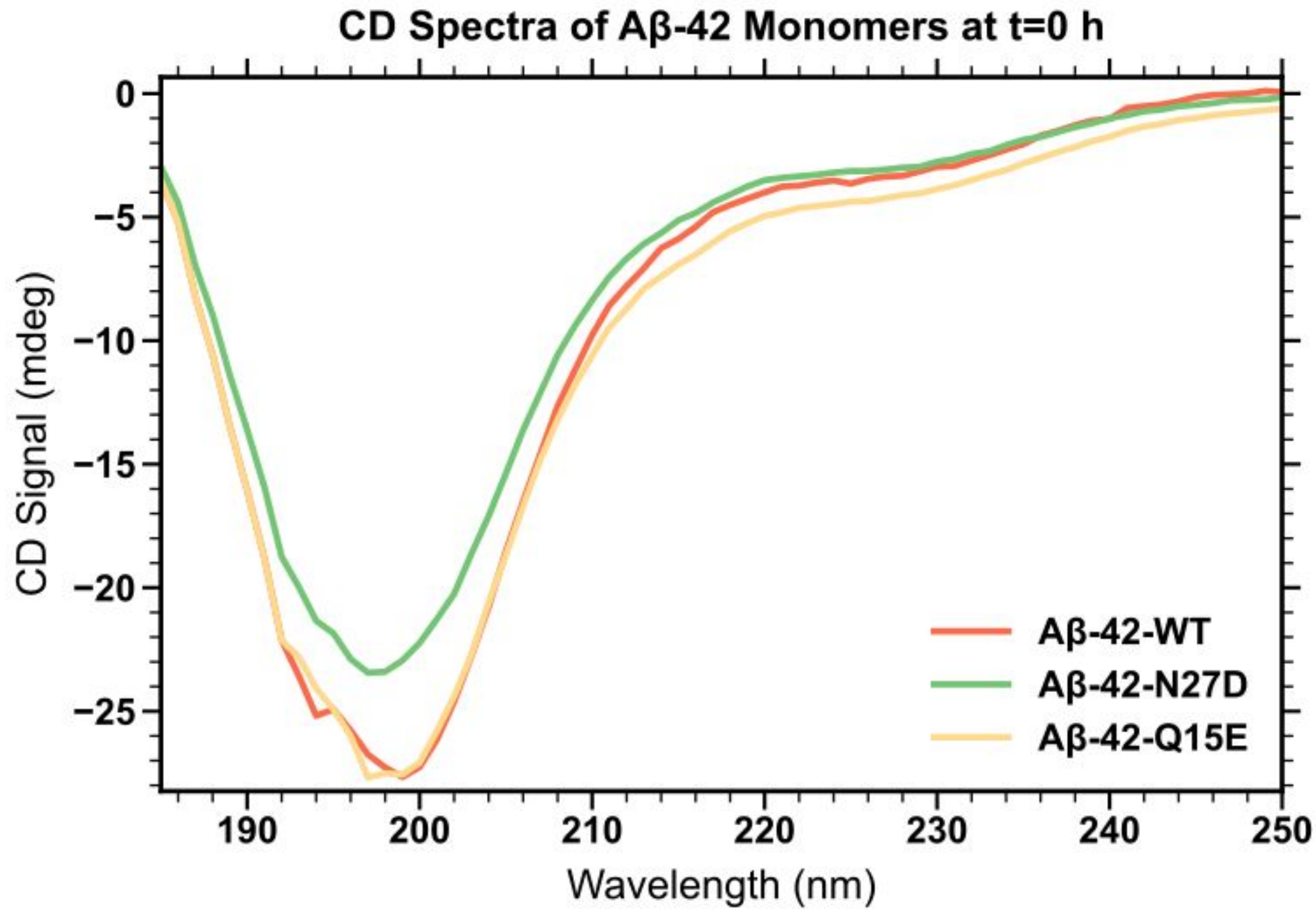

Supporting Figure 3. Far-UV CD spectra (250–185 nm, 25 °C; 1 mm path, 5 replicates averaged) of the freshly SEC-isolated monomers of Aβ-42-WT, Aβ-42-Q15E, and Aβ-42-N27D at t = 0 h. All three proteoforms display a single dominant minimum near 200 nm with no 218 nm minimum, consistent with a random-coil conformation.

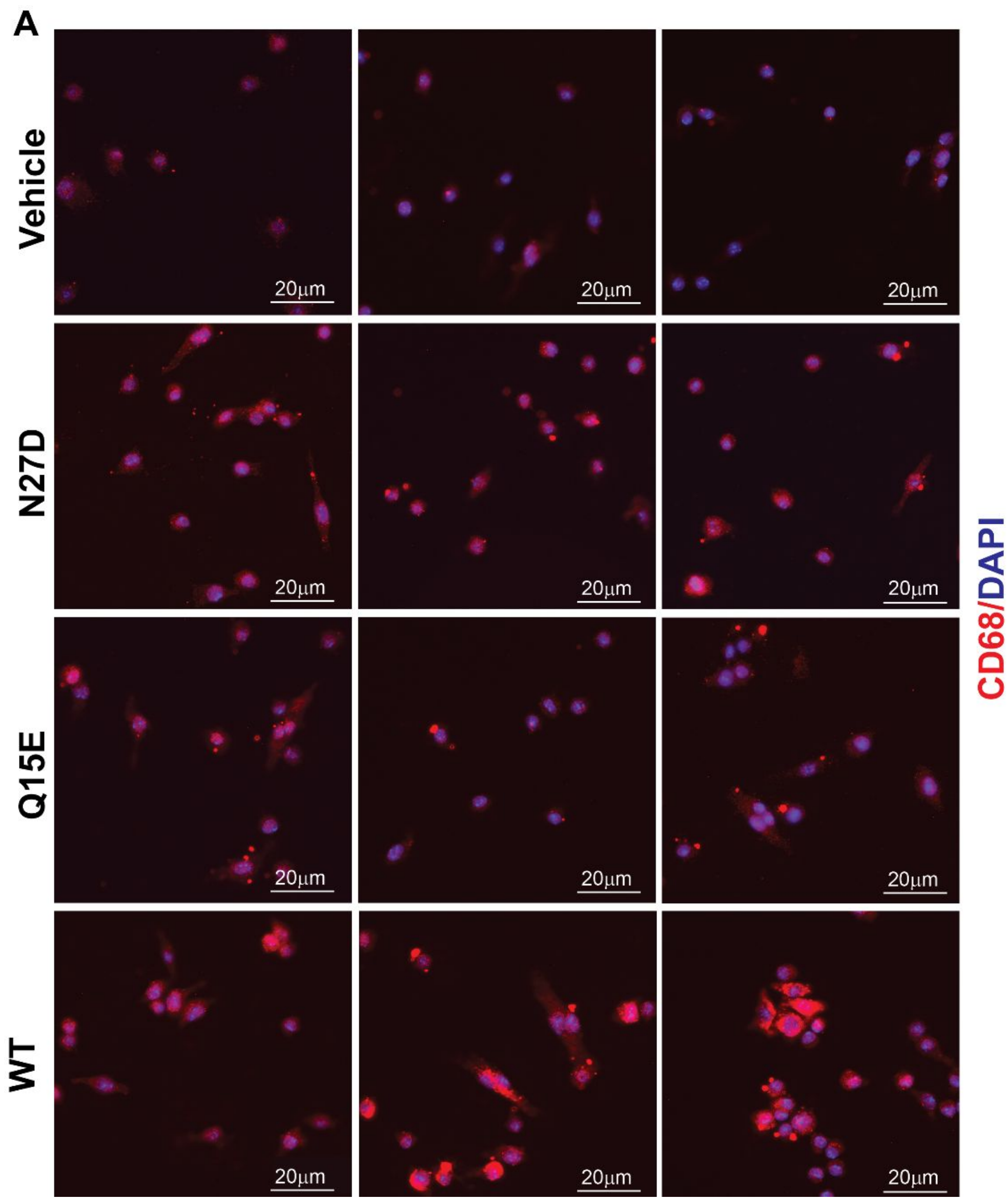

Supporting Figure 4. Deamidation abrogates Aβ-induced CD68 expression in mouse microglial cell line. A. Representative ICC for CD68 (red) and DAPI (blue) after 2 hours of incubation with 50 nM of each Aβ variant (scale bar: 20 µm, representative sections from n = 12-13 sample well/group).

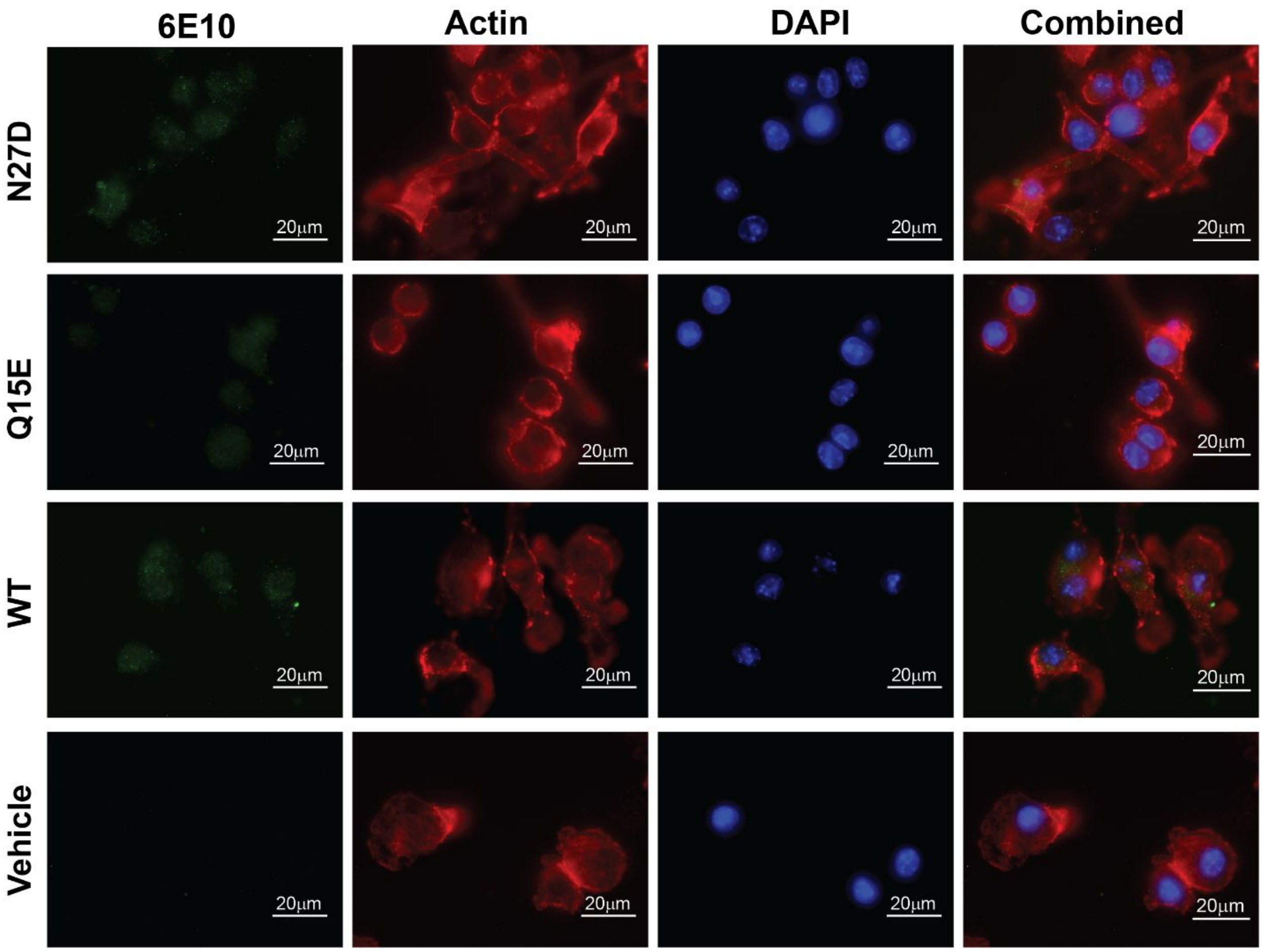

Supporting Figure 5. Deamidation of Aβ-42 results in diminished internalization at 2 hours post treatment. ICC for 6E10 (green), F-actin (red), and DAPI (blue) after 2 hours of incubation with 50 nM of each Aβ variant (scale bar: 20 μm, representative sections from n = 6 sample well/group).

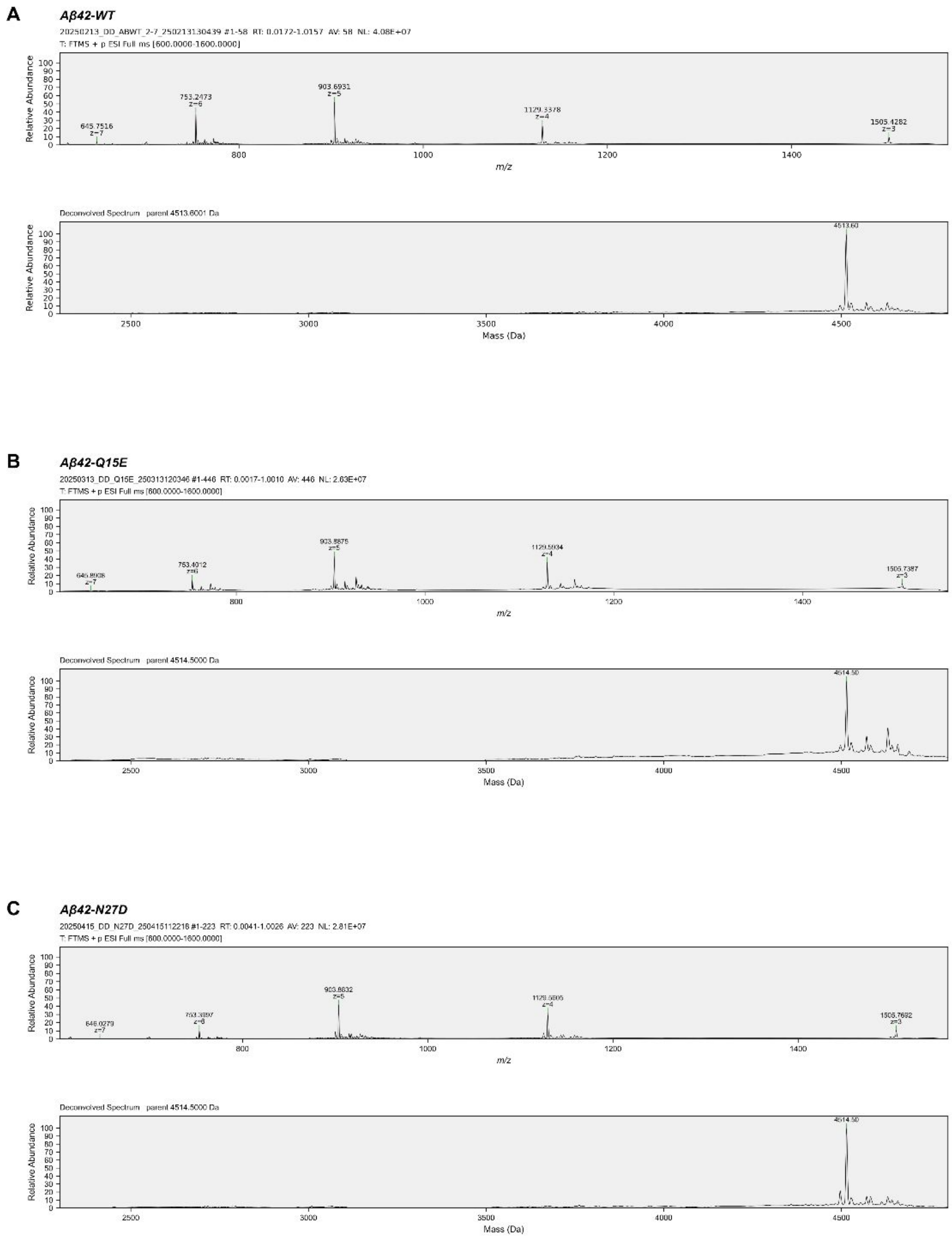

Supporting Figure 6. ESI-MS verification of synthesized Aβ-42 peptides. Each panel shows the averaged charge-state spectrum (top) and UniDec-deconvoluted neutral mass (bottom) for (**A**) Aβ42-WT, (**B**) Aβ42-Q15E, and (**C**) Aβ42-N27D. Deconvoluted parent masses: 4513.6, 4514.5, and 4514.5 Da. The +1 Da shift of Q15E and N27D relative to WT is consistent with the Gln → Glu and Asn → Asp substitutions.
